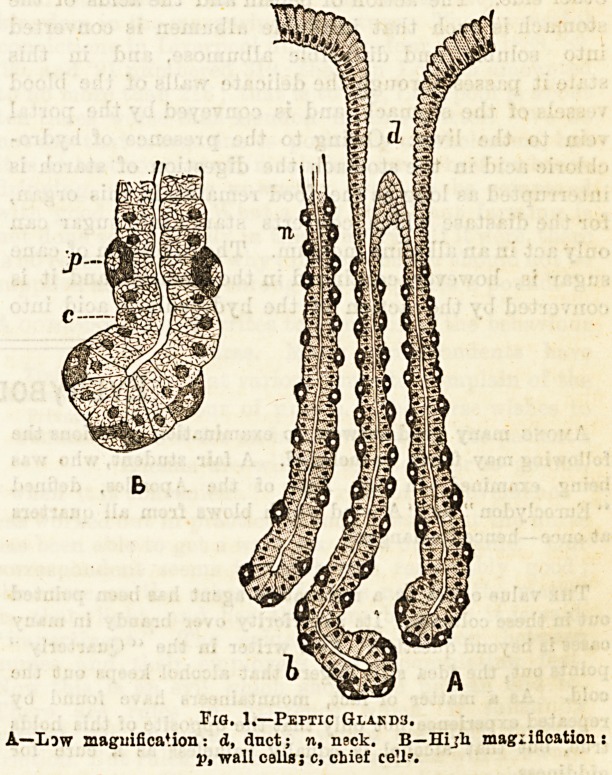# Diet in Disease

**Published:** 1892-12-03

**Authors:** Ernest Hart

**Affiliations:** Bachelier-ès-Sciences-es-Lettres (rèstreint), formerly Student of the Faculty of Medicine of Paris, and of the London School of Medicine for Women


### DIET IN DISEASE.

BY MRS. ERNEST HART, Bachelier-ès-Sciences-es-Lettres (rèstreint), formerly Student of the Faculty of Medicine of Paris, and of the London School of Medicine for Women.

### VIII.—DIGESTION.

HAVING now considered the nature and constitution of the various foods which sustain and build up the body, I will proceed to describe the methods and processes by which they are digested and assimilated.

**Digestion of Food in the Mouth.**—When solid food is placed in the mouth, it is masticated or ground by the molar teeth. It is at the same time thoroughly mixed with the saliva, which is poured out in abundance at the moment required, by the salivary glands the ducts of which open into the mouth on the inner side of the cheeks and under the tongue. The period of time that the food remains in the mouth subject to mastication and the influence of the saliva varies with different individuals; but it is well that this period should be as long as possible, in order that the food may be completely broken up, and the tougher and harder portions rendered fit for digestion in the stomach. Besides mastication, the first step in the digestion of *starch* takes place in the mouth.

**Starch** is contained in a great number of the vegetable foods which are common articles of diet—namely, potatoes, flour, peas, beans, &c. In its uncooked condition, it is incapable of digestion by man. It exists in the form of small granules, composed of concentric layers of material. These granules are insoluble in cold water, but on being boiled or placed in hot water their outer envelope bursts, and the contents swell up, the whole forming an opalescent gelatinous mass. In order that starch may be made perfectly soluble, so as to pass through the coats of the minute blood-vessels of the intestines, it is necessary for it to be converted into sugar, and one, therefore, of the most important acts of digestion is the conversion of starch into sugar. This is brought about by the action of a ferment or diastase. Such a ferment or diastase is present in the saliva, and is called *ptyalin*, which, acting on the starch in the food, partially converts it into sugar while mastication is going on. The mouthful of food having been thoroughly ground by the action of the molar teeth or grinders, and well mixed with the saliva, it is rolled into a ball or bolus by the tongue, and passed, by the act of swallowing, to the back of the mouth. It is here seized by the self-acting or involuntary muscles which form the pillars of the throat, and it is passed by their action, and by the rolling upwards and backwards of the root of the tongue over the epiglottis or trap-door which closes the opening into the windpipe, into the gullet or œsophagus, a long tube which conducts to the stomach.

**Digestion in the Stomach.**—The stomach is a large, hollow, bag-like organ, larger at one end than the other, and furnished with strong muscular walls which can contract in every direction. It is lined inside with a highly organised mucous membrane. This mucous membrane consists of follicles or glove-like depressions, some of which are simple, others divided or branched. The glands of the stomach are of two kinds, *mucous glands*, which are lined with large, clear, rounded cells, which almost entirely fill up the central opening of the tube, and *peptic glands*, which contain large spheroidal and finely granular cells. (See fig. 1.) It is these cells which are supposed to be principally concerned in the secretion of pepsin. The result of the action of the two kinds of glands in the stomach is that a mucous fluid containing pepsin, and called the gastric juice, is abundantly poured out at the moments of digestion. By means of the slow, continuous, and churning action of the stomach, the food is constantly rolled from one end to the other and becomes thoroughly mixed into a fluid pulp or juice. Unlike all the other digestive fluids, the *gastric juice is acid.* It must be remembered, and it will be found to be very important to bear in mind, when considering later the question of dyspepsia and its treatment by diet, that there are three chief ingredients of the gastric juice,

FIG. 1.—PEPTIC GLANDS.

A—Low magnification: *d*, duct; *n*, neck. B—High magnification: *p*, wall cells; *c*, chief cells.

namely, pepsin, free acid, and mucus, all of which are necessary in the process of gastric digestion.

The peculiar quality of the pepsin is that it has the power of digesting and dissolving substances of an albuminous nature; the mucus seems to dilute the pepsin, and to prevent it from acting too violently, even on the coats of the stomach itself, and the free acid—which is hydrochloric acid—is necessary in order to enable the pepsin to act, for it is only in the presence of a free acid that pepsin is operative. Hydrochloric acid has also an antiseptic action, by stopping abnormal fermentation and by destroying the numerous bacilli and minute organisms swallowed with the food, which would otherwise flourish in the stomach and give rise to active fermentation.

**The Digestion of Albuminous Substances.**—A large part of the food is necessarily composed of albuminous substances. They form, as I have shown the chief constituents of meat, cheese, milk, and eggs, and are found in many vegetable foods, such as peas, beans, lentils, and also in wheat and oats. In the condition in which albumen is introduced into the stomach it is incapable of being absorbed by the blood vessels. It must, therefore, first be brought into such a condition that it will pass easily through the coats of the veins and be introduced into the circulation. That albumen in its usual condition will not pass through an animal membrane may be proved by placing the white of an egg in a bladder tightly stretched over a vase quite full of water. The white of egg, which is fine albumen, will not pass through the bladder into the water. If, however, some pepsin and a free acid be added, and the whole allowed to stand at a temperature of about 100 deg., the albumen will undergo such changes that it will pass easily through the bladder, and be found diffused in the water on the other side. The action of pepsin and the acids of the stomach is such that insoluble albumen is converted into soluble and diffusible albumose, and in this state it passes through the delicate walls of the blood vessels of the stomach, and is conveyed by the portal vein to the liver. Owing to the presence of hydrochloric acid in the stomach the digestion of starch is interrupted as long as the food remains in this organ, for the diastase which converts starch into sugar can only act in an alkaline medium. The digestion of cane sugar is, however, continued in the stomach, and it is converted by the action of the hydrochloric acid into glucose or grape sugar, in which state it is readily absorbed by the blood vessels.

The time occupied by gastric digestion varies from three to four hours. Some articles of food take much longer to digest than others. In arranging the diet of a dyspeptic, it is important to know which foods are most quickly and easily digested in the stomach. The process of digestion in the stomach being completed, the albumen being turned into soluble albumose, the cane sugar into glucose, and a large part of these substances having been absorbed direct by the blood vessels which ramify on the surface of the stomach, the semi-fluid mass passes gradually, and in small quantities at a time, out of the stomach through the narrow opening of the "pylorus." The pylorus is a small circular passage or opening, which is closed by strong encircling muscular fibres during the process of gastric digestion. If the chyme, a partially digested mass, is thoroughly well mixed, and there are not any large undigested or irritating portions present, the food passes through the pylorus without any feeling of discomfort. If, however, portions of food are undigested, the pylorus may refuse to let the chyme pass, and the muscles of the stomach being then thrown sympathetically into a state of irritation, may contract spasmodically, and the food be ejected forcibly from the mouth by the act of vomiting. If, on the other hand, the stomach has well performed its part, the food passes into the duodenum.

Articles of this series have appeared as follows': I. and II. Food and Food Values, October 1st and 8th; III. Stimulants and Alcohol, October 15th; IV. Stimulants: The Coca of Peru, Coca Wine, October 22nd; V. Restoratives: Tea, November 5th; VI. Restoratives: Coffee, Cocoa, and Chocolate, November 19th; VII. Water, Salts, November 26th.

## Figures and Tables

**Fig. 1. f1:**